# Active Smoking Increases Microsomal PGE_2_-Synthase-1/PGE-Receptor-4 Axis in Human Abdominal Aortic Aneurysms

**DOI:** 10.1155/2014/316150

**Published:** 2014-04-30

**Authors:** Jaime-Félix Dilmé, David Solà-Villà, Sergi Bellmunt, José-María Romero, José-Román Escudero, Mercedes Camacho, Luis Vila

**Affiliations:** ^1^Angiology, Vascular Biology and Inflammation Laboratory, Institute of Biomedical Research of Hospital Santa Creu i Sant Pau (II-B Sant Pau), 08025 Barcelona, Spain; ^2^Vascular Surgery Department, Institute of Biomedical Research of Hospital Santa Creu i Sant Pau (II-B Sant Pau), 08025 Barcelona, Spain; ^3^Autonomous University of Barcelona, Institute of Biomedical Research of Hospital Santa Creu i Sant Pau (II-B Sant Pau), 08025 Barcelona, Spain

## Abstract

*Background*. The cyclooxygenase- (COX-) 2/microsomal PGE-synthase- (mPGES-) 1/PGE-receptor- (EP-) 4 axis could play a key role in the physiopathology of abdominal aortic aneurysm (AAA) in humans. In this study, we investigated the influence of cardiovascular risk factors on the expression of the PGE_2_ pathway in human AAA. *Methods*. Aortic (*n* = 89) and plasma (*n* = 79) samples from patients who underwent AAA repair were collected. Patients were grouped according to risk factors. COX-isoenzymes, mPGES-1, EPs, **α**-actin, and CD45 and CD68 transcripts levels were quantified by QRT-PCR and plasma PGE_2_ metabolites by EIA. *Results*. Current smoking (CS) patients compared to no-CS had significantly higher local levels of mPGES-1 (*P* = 0.009), EP-4 (*P* = 0.007), and PGE_2_ metabolites plasma levels (*P* = 0.008). In the multiple linear regression analysis, these parameters remained significantly enhanced in CS after adding confounding factors. Results from association studies with cell type markers suggested that the increased mPGES-1/EP-4 levels were mainly associated with microvascular endothelial cells. *Conclusions*. This study shows that elements of the PGE_2_ pathway, which play an important role in AAA development, are increased in CS. These results provide insight into the relevance of tobacco smoking in AAA development and reinforce the potential of mPGES-1 and EP-4 as targets for therapy in AAA patients.

## 1. Introduction

Abdominal aortic aneurysm (AAA) affects a high percentage of the aged population in industrialized countries and mortality rates associated with rupture of AAA are high. The aetiology of AAA is essentially unknown, but it is generally accepted that environmental and genetic factors contribute substantially to the risk of AAA [1]. An association between AAA and atherosclerosis has long been recognized. There are, however, histological and epidemiologic differences between the two diseases. The major risk factors for AAA are increasing age, family history, male gender, hyperlipidemia (HL), arterial hypertension (HTN), and smoking [1]. Of these, smoking seems to be the most relevant, with a considerably higher risk for AAA than for atherosclerosis [2]. Diabetes mellitus (DM) is a well-established risk factor for atherosclerosis and has been reported to be protective for AAA [3]. Studying the influence of cardiovascular risk factors, particularly those that differentially influence AAA and atherosclerosis, could help to ascertain the causes of AAA.

Human AAA is characterized by leukocyte infiltration into adventitia and media and depletion of vascular smooth muscle cells (VSMC) in the media. Another relevant feature of the disorder is a decrease in AAA wall strength caused by the breakdown of elastic fibres and the hypervascularization of aortic tissue [4]. A key factor in the pathophysiology of AAA is PGE_2_. PGE_2_ induces expression of metalloproteinases and inhibits the production of macromolecules of the extracellular matrix [5, 6]. It acts as a signalling molecule in response to proangiogenic factors and also induces release of several angiogenic factors in a manner of positive loop [7–10]. Consequently, inhibition or deletion of the enzymes involved in the biosynthetic pathway of PGE_2_ interferes in AAA development in animal models [11–15].

PGE_2_ biosynthesis begins with the formation of PGH_2_ from arachidonic acid (AAc) catalyzed by cyclooxygenase (COX). PGH_2_ is in turn isomerized to PGE_2_ by PGE-synthases (PGES) [16]. The microsomal isoform of PGES, mPGES-1, is inducible by proinflammatory cytokines and it seems to be the essential PGES isoenzyme involved in PGE_2_ biosynthesis under inflammatory conditions [17–19]. COX-2/mPGES-1 is widely regarded as the main contributing enzymatic tandem for PGE_2_ biosynthesis under pathological conditions.

PGE_2_ exerts its cellular effects by binding to four distinct E-prostanoid receptors (EP1–4). EPs belong to the family of seven transmembrane G protein-coupled rhodopsin-type receptors. Each receptor has different and often opposing biological effects. EP2 and EP4 are Gs proteins-coupled receptors and they upregulate intracellular cAMP levels. EP3 usually counteracts EP2- and EP4-mediated upregulation of cAMP by preferentially coupling to Gi proteins [20]. Also, interventions on the PGE_2_ receptor display benefits in experimental AAA [21, 22].

We recently reported that COX-2, mPGES-1, EP-2, and EP-4 are upregulated in AAA and that the COX-2/mPGES-1/EP-4 axis could play a relevant role in AAA-associated hypervascularization from the early stages of AAA development [23]. However, information is lacking regarding the influence of cardiovascular risk factors on local levels of the COX-2/mPGES-1/EP-4 axis in AAA.

In this study, we aimed to evaluate the influence of major cardiovascular risk factors on the PGE_2_ pathway in human abdominal aortic aneurysms.

## 2. Methods

### 2.1. Patients

The inclusion criteria for this study were patients who underwent open repair for AAA with an atherosclerotic aneurysm and in whom an infrarenal aorta biopsy was taken during the intervention. The exclusion criteria were absence or inadequate aortic biopsy, pseudoaneurysms, and infectious or inflammatory aneurysms. All patients underwent surgery at Hospital de la Santa Creu i Sant Pau (HSCSP). The study was approved by the local ethics committee, and informed consent was obtained from each patient. All procedures were reviewed by the institutional review board at HSCSP. The investigation conforms with the principles outlined in the Declaration of Helsinki.

### 2.2. Tissue Samples

Samples were obtained from remaining midinfrarenal aortic wall after exclusion and prosthetic replacement of AAA. When luminal thrombus was present it was separated before the aorta biopsy was taken and aortic tissue was washed twice with cold phosphate buffered saline (PBS). A portion of each sample was placed in RNAlater solution (Qiagen GmbH, Hilden, Germany) and stored at 4°C for 24 hours before long-term storage at −80°C until further processing for RNA isolation. When possible a portion was fixed in formalin solution 10% (Sigma-Aldrich, Inc., St. Louis, MO) for 24 h and included in paraffin for immunohistochemical studies.

### 2.3. Risk Factors Definitions

The risk factors definitions used in this study were diabetes mellitus (DM): glycated haemoglobin >5.8% or use of oral antidiabetic drugs or insulin; arterial hypertension (HTN): systolic blood pressure ≥140 mm Hg, diastolic blood pressure ≥80 mm Hg, or use of antihypertensive medication; hyperlipidemia (HL): a total cholesterol >6.2 mmol/L, LDL cholesterol >1.70 mmol/L, or triglycerides >1.65 mmol/L; smoking: categorized into 2 groups: current smoking (CS): smokers and ex-smokers who stopped smoking <1 year and noncurrent smoking (N-CS): never-smokers and ex-smokers who stopped smoking >1 year; chronic occlusive pulmonary disease (COPD): FEV1/FVC < 0.7; and renal insufficiency (RI): estimated glomerular filtration rate (eGFR) ≤60 mL/min/1.73 m^2^ calculated using the Chronic Kidney Disease Epidemiology Collaboration (CKD-EPI) equation [24].

### 2.4. Analysis of mRNA Levels in the Tissues and Culture Cells

Tissues were homogenized in the FastPrep-24 homogenizer and Lysing Matrix D tubes (MP Biomedicals, Solon, OH). RNA was extracted using Trizol (Invitrogen, Carlsbad, CA) following the manufacturer's instructions. cDNA was prepared by reverse transcription of 1 *μ*g RNA using High-Capacity cDNA Archive kit with random hexamers (Applied Biosystems, Foster City, CA). mRNA expression of the selected genes was studied by real-time PCR in an ABI Prism 7900HT using predesigned validated assays (TaqMan Gene Expression Assays; Applied Biosystems) and universal thermal cycling parameters. Relative expression was expressed as transcript/*β*-actin ratios.

### 2.5. Plasma Levels of PGE_2_

A sample of 10 mL of peripheral blood was collected in heparin-containing tubes. Blood was collected before anesthesia in the operating room. It was centrifuged immediately and plasma aliquoted and frozen at −80°C until analysis. PGE_2_ and 13,14-dihydro-15-oxo-PGE_2_ were determined using enzyme immunoassay (EIA) kits (Cayman Chemical, Ann Arbor, MI) following the manufacturer's instructions.

### 2.6. Immunohistochemistry

Immunohistochemical studies were performed using a rabbit polyclonal antibody against mPGES-1 (ref. HPA045064 prestige antibodies, diluted 1 : 50) from Sigma and a mouse monoclonal antibody anti-EP-4 (ref. 101775, diluted 1 : 100) from Cayman Chemical. Blanks were performed using the corresponding blocking peptides all from Cayman. Monoclonal antibodies (ref. M0616, diluted 1 : 35; ref. IR751 and ref. IR613, without further dilution) from Dako were used for von Willebrand Factor (vWF, endothelial cell marker), CD45 (pan-leukocyte marker), and CD68 immunostaining. Three-micrometer sections of paraffin-embedded tissue samples were stained in a Dako Autostainer Link 48 using the Dako EnVision Flex kit. Diaminobenzidine was used as chromogen. Immunostainings used for comparative purposes were processed simultaneously.

### 2.7. Statistical Analysis

SPSS and Sigma-Stat software were used for statistical analysis. All data regarding transcript levels are expressed relative to *β*-actin ×1000. All quantitative data in this study were nonnormally distributed and expressed as median (25th–75th percentile). We used the Mann-Whitney rank sum test to compare the two groups. The Pearson product moment correlation was used to evaluate the association between continuous variables after Log10 transformation of data with nonnormal distribution. Data were also analyzed using a multiple linear model including the effect of the identified risk factors and controlling possible confounding factors; a logarithmic transformation was applied to those outcomes without a normal distribution. A *P* value below 0.05 was considered significant.

## 3. Results

From 2008 to 2012, 225 elective AAA were performed in our center (Department of Vascular and Endovascular Surgery of the Hospital de la Santa Creu i Sant Pau in Barcelona, Spain). 166 patients underwent open surgery and 59 had endovascular aortic repair (EVAR). An adequate biopsy was obtained from 89 patients during the intervention.

Table 1 shows the number and characteristics of participants. None of the risk factors caused significant differences in either COX-1 or COX-2 levels between AAA samples (Table 2). Regarding mPGES-1, only current smoking (CS) showed significant high transcript levels when compared with the noncurrent smokers (N-CS) group. mPGES-1 expression was higher in patients suffering COPD than in those without COPD, but the difference did not reach statistical significance. Next we evaluated the association between risk factors. Following *χ* ^2^ analysis, we identified COPD and HTN as variables likely related to CS. As these two variables could act as confounding factors (Table 3), we applied multiple linear regression analysis. The relationship between CS and mPGES-1 remained stable even in the presence of the confounding factors (COPD and HTN) in the statistics (*P* = 0.03). We found that plasma levels of PGE_2_ in AAA patients were significantly enhanced in the CS group when compared with the N-CS group (Table 4). None of the other risk factors analysed had statistically significant differences regarding circulating levels of PGE_2_. When confounding variables were taken into account, multiple linear regression analysis showed statistically significant differences between CS and N-CS groups (*P* = 0.012).

**Figure 1:**
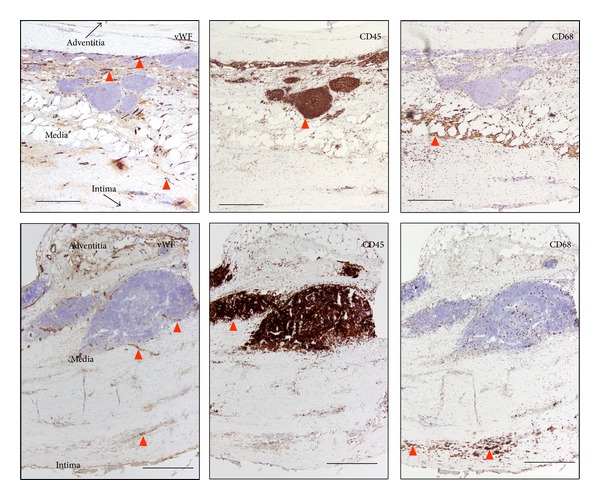
Representative immunohistochemical images of vWF (endothelial cell marker), CD45 (pan-leukocyte marker), and CD68 (macrophage marker) in aorta samples from two AAA patients. Red arrow-ends indicate some immunostained cells. Size bars: 500 *μ*m. In upper panels black-tipped arrows are used to orient the position of the vessel by indicating to where intima and adventitia are.

**Figure 2:**
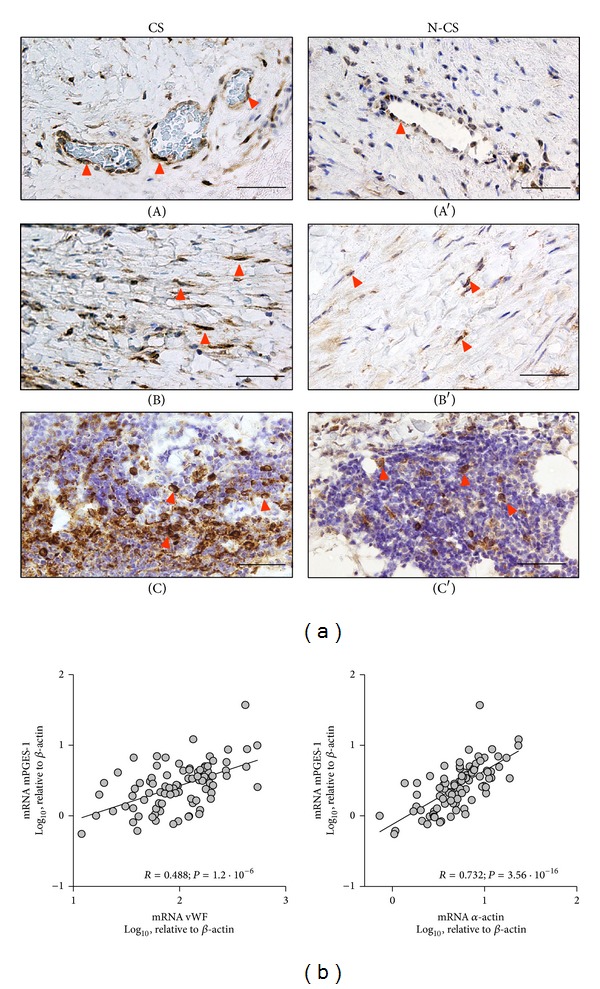
(a) Representative immunohistochemical images of mPGES-1 in aorta samples from current smokers (CS, left) and noncurrent smokers (N-CS, right) patients, showing mPGES-1 immunostaining of MVEC (A, A′), VSMC of the media layer (B, B′), and mPGES-1 positive leukocytes (C, C′). Red arrow-ends indicate some immunostained cells. Size bars: 50 *μ*m. (b) Correlation between mPGES-1 transcript levels and those of vascular cell markers; Pearson product moment correlation applied to log 10-transformed data (*n* = 89).

Figure 1(a) shows examples of leukocyte infiltration of AAA samples. Microvessels were abundant in the adventitia and in the media. While major infiltrated leukocytes (determined by CD45 immunostaining) systematically accumulated in perivascular areas of microvessels, macrophages (determined by CD68 immunostaining) displayed a more scattered pattern. Immunohistochemistry study of mPGES-1 was performed in AAA samples from 4 patients of each smoking group to localize the protein expression. No differences were observed between CS and N-CS groups regarding mPGES-1 protein location. mPGES-1 was located in MVEC, VSMC, and infiltrating leukocytes (Figure 2(a)). In an attempt to approach the origin of the differences of mPGES-1 in our AAA samples, we determined the statistical association of mPGES-1 transcript levels with cell-characteristic markers as independent variables. We analysed mRNA levels of von Willebrand Factor (vWF), *α*-actin, and CD68 as markers of endothelial cell, VSMC, and macrophages, respectively. No significant statistical association was found between mPGES-1 and CD68 transcript levels (not shown). In contrast, mPGES-1 levels significantly correlated with both *α*-actin and vWF (Figure 2(b)). We then explored the influence of CS on the cell markers and no significant differences were found between CS and N-CS (not shown).

**Figure 3:**
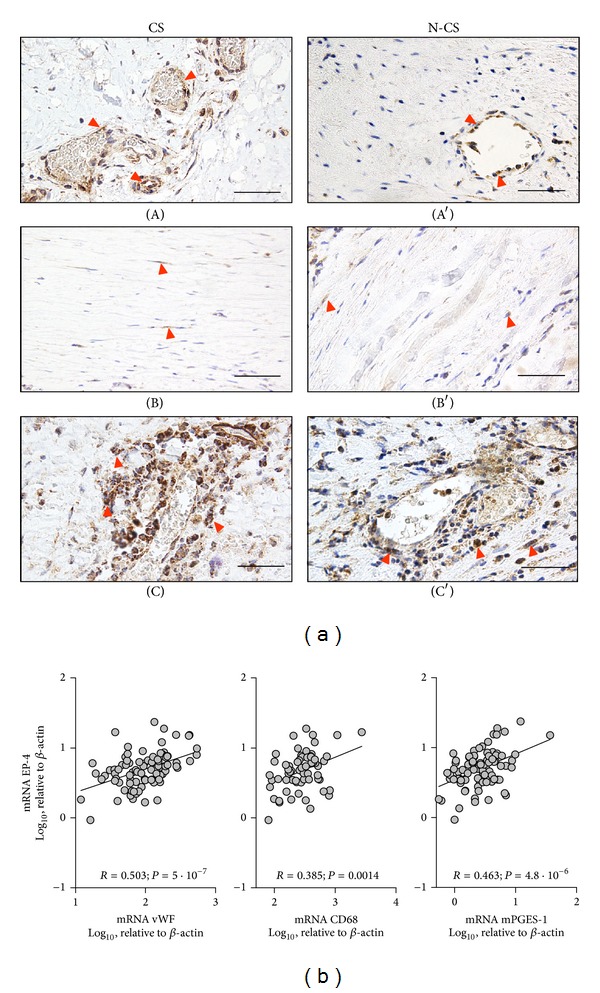
(a) Representative immunohistochemical images of EP-4 in aorta samples from current smokers (CS, left) and noncurrent smokers (N-CS, right) patients, showing EP-4 immunostaining of MVEC (A, A′), the scarce presence of EP-4 immunostaining in media layer in areas free of leucocyte infiltration (B, B′), and EP-4 positive leukocytes (C, C′). Red arrow-ends indicate some immunostained cells. Size bars: 50 *μ*m. (b) Correlation between mPGES-1 transcript levels and those of endothelial cell and macrophage markers and between EP-4 and mPGES-1; Pearson product moment correlation applied to log 10-transformed data (*n* = 89).

Regarding PGE_2_ receptors, EP-1 was expressed only scarcely in AAA samples and therefore was not considered for the analysis of cardiovascular risk factors. No risk factor caused statistically significant variation of either EP-2 or EP-3. In contrast, significant differences between CS and N-CS groups were found regarding EP-4, CS having the highest transcript levels when simple statistical analysis was applied (Table 5). Moreover, when confounding variables (COPD and HTN) were introduced in statistics the relationship between CS and EP-4 remained stable (*P* = 0.007). EP-4 was almost undetectable in VSMC in culture in terms of mRNA (78-fold lower than in MVEC, not shown). Immunohistochemical staining indicated that EP-4 was much less expressed in VSMC than in MVEC. EP-4 was detected mainly in MVEC and infiltrating leucocytes. No differences between CS and N-CS groups were observed regarding localization of EP-4 (Figure 3(a)). The association between EP-4 and CD68 transcript levels was poor (Figure 3(b)), whereas the association with vWF was stronger. In addition, we observed an excellent correlation between mPGES-1 and EP-4 transcript levels.

## 4. Discussion

This is the first study to describe the influence of cardiovascular risk factors on local levels of PGE_2_ pathway in abdominal aortic aneurysm. We found that current smoking was associated with increased local expression of transcript levels of mPGES-1 in AAA.

In accordance with other reports, we recently found that local expression of COX-2 was increased in aneurismal tissue. Additionally, we reported that mPGES-1 was increased in AAA and that the upregulation of COX-2/mPGES-1 precedes maximal leukocyte infiltration [23]. We found that EP-2 and EP-4 were upregulated in AAA, whereas EP-3 was significantly downregulated. An interesting finding was that PGE_2_-mediated in vitro angiogenesis was fully dependent on EP-4. Herein, we report the influence of the most relevant cardiovascular risk factors on the COX-2/mPGES-1/EP-4 axis expression.

Current smoking was the only cardiovascular risk factor that was significantly associated with increased local expression of mPGES-1 in aneurysmatic aorta. However, it had no effect on COX-2 expression. This observation is consistent with the fact that COX-2 and mPGES-1 display different regulation in vitro [18, 19]. The increase in the expression of PGE_2_ biosynthetic machinery was consistent with the higher levels of PGE_2_ observed in the plasma of the smoker patients. Nevertheless, as AAA should be considered a systemic disease of the vasculature [1], other vascular territories may contribute to circulating levels of PGE_2_.

The presence of mPGES-1 in a particular cell is necessary for PGE_2_ biosynthesis [17–19, 25]. All vascular cells [17, 23, 26] and leukocytes [23], likely mainly macrophages, express mPGES-1. Our present results show that the expression levels of mPGES-1 correlate with the expression of vascular cell markers rather than with infiltrating macrophages markers suggesting that mPGES-1 is expressed in VSMC and MVEC as we reported before [17, 18, 26]. Therefore, changes in the number of VSMC and MVEC influence mPGES-1 levels. The absence of statistically significant differences between the CS and N-CS groups regarding transcript levels of vascular cell markers, together with increased levels of mPGES-1 in the CS group, strongly suggests that expression of mPGES-1 is effectively upregulated in vascular cells in CS group. Nevertheless, VSMC tends to be reduced whereas MVEC are substantially increased in AAA [1, 4, 26]. Altogether, our results heighten the role that MVEC-associated mPGES-1 and current smoking could have in AAA progression. Of course this does not rule out the important role of macrophages in AAA progression. Indeed, macrophage COX-2-derived PGE_2_ has been found to be relevant in the pathogenesis and rupture of AAA [12, 27, 28].

Regarding PGE_2_ receptors, only CS had an effect on the local levels of EP-4. Active smokers had significantly higher levels of EP-4 transcript. EP-4 is the most abundant subtype of PGE_2_ receptor in endothelial cells and it is a key receptor for PGE_2_-induced angiogenesis [23]. It was expressed in leukocytes, but we found that variation of EP-4 expression correlated better with vWF mRNA levels (endothelial cell marker) than with CD68 (macrophage marker). The statistical significance of the difference between CS and N-CS regarding mPGES-1/EP-4 axis was maintained after the introduction of HTN and COPD as confounding variables in the statistics. Moreover, the association of mPGES-1 and EP-4 transcript levels was highly significant. Taken together, our results support the concept that smoking substantially affects aortic vascular cells and suggest that MVEC are particularly affected.

Smoking is a particularly relevant risk factor for AAA [29]. It has been reported that the association between smoking and AAA is 2.5-fold higher than the association between smoking and coronary heart disease [2]. Tobacco smoke also enhances AAA formation in animal models [30, 31]. Our data indicated that mPGES-1/EP-4 is mainly linked to vascular cell state, whereas local levels of COX-2 depend on several factors such as the inflammatory infiltrate in the aortic wall. Local levels of COX-2 were not associated with any particular marker. Therefore, it seems that smoking affects the state of the vascular cells more than the degree of leukocyte infiltration. Many reports provide evidence supporting the association between renal function and atherosclerosis, and eGFR is an independent prognostic factor for cardiovascular disease [32]. We recently reported that eGFR is an excellent predictor of vascular events in patients with peripheral arterial disease [33]. Since RI could indicate the atherosclerosis level of patients, our results suggest that the effect of smoking on local levels of mPGES-1/EP-4 is independent of the atherosclerosis and COPD states of patients with AAA. These results support the lack of association of COPD and AAA with smoking described in a previous report [34]. More research is needed to ascertain the molecular species and signalling pathways involved in the effect of tobacco smoke on the induction of mPGES-1 and EP-4 expression in human AAA.

In conclusion, we show for the first time that current smoking increases MVEC-associated mPGES-1/EP-4 and provides further insight into the relevance of tobacco smoking in AAA development. Our data are consistent with reports showing that suppression of either mPGES-1 or EP-4 expression reduces AAA development in animal models [15, 21, 22] and reinforce the potential of mPGES-1 and EP-4 as alternative targets for therapy in AAA patients.

## Figures and Tables

**Table 1:** Clinical characteristics of individuals with AAA included in the study, demographics, and risk factors.

Measurements characteristic	mRNA	Plasma levels
Number	89	79
Aortic diameter (mm)	66 ± 12.7	64.3 ± 11.6
Age (years)	71.1 ± 6.5	72.4 ± 6.8
Male	87 (97.8%)	75 (94.9%)
Diabetes mellitus	19 (21.3%)	21 (26.6%)
Hypertension	63 (70.8%)	60 (75.9%)
Hyperlipidemia	54 (60.7%)	51 (64.6%)
Smoking habits		
Noncurrent smokers	63 (70.8%)	60 (75.9%)
Current smokers	26 (29.2%)	19 (24.1%)
Coronary artery disease (CAD)	20 (22.4%)	22 (27.9%)
Angor pectoris	2 (2.2%)	4 (5.1%)
Myocardial infarction	6 (6.7%)	6 (7.6%)
Coronary intervention/CABG	12 (13.5%)	12 (15.2%)
Chronic renal insufficiency	34 (38.2%)	23 (29.1%)
Dialysis	0	0
Peripheral vascular disease	48 (53.9%)	45 (57%)
Absence pulses	31 (34.8%)	30 (38%)
Intermittent claudication	17 (19.1%)	15 (19%)
Cerebrovascular disease	7 (7.9%)	7 (8.8%)
Cerebral vascular attack	4 (4.5%)	5 (6.3%)
Transient ischemic attack	3 (3.4%)	2 (2.5%)
COPD	26 (29.2%)	17 (21.5%)
Antiplatelet users	47 (53.4%)	48 (61.5%)
Statins users	57 (64%)	58 (73.4%)
ACEIs users	23 (20.7%)	24 (30.8%)
NSAD users	6 (6.7%)	3 (3.8%)
Corticoid users	6 (6.7%)	4 (5.1%)
Immunosuppressors	4 (3.5%)	2 (2.5%)

Nominal variables are presented as number and as percentage (%) and continuous variables as mean ± SD. Aortic diameter: aneurysm maximum transverse diameter in mm; chronic renal insufficiency: estimated glomerular filtration rate (eGFR) <60 mL/min/1.73 m^2^; noncurrent smokers: never smokers or quit smoking >1 year; CABG: coronary artery bypass grafting; COPD: chronic occlusive pulmonary disease.

**Table 2:** Transcript levels of COX isoenzymes and mPGES-1 in the AAA samples stratified by cardiovascular risk factors. The number of patients in every group is indicated in Table 1. Data are expressed as relative to *β*-actin x1000.

	Median	25th–75th percentile	*P* value versus no-factor^a^
COX-1			
No current smoking	10.7	7.68–14.3	
Current smoking	11.6	9.47–18.7	0.109
No renal insufficiency	10.9	8.50–16.3	
Renal insufficiency	10.7	7.78–15.8	0.896
No hypertension	10.8	9.62–16.1	
Hypertension	10.7	7.49–15.8	0.419
No diabetes mellitus	11.0	8.67–15.9	
Diabetes mellitus	9.71	6.48–15.1	0.365
No hyperlipidemia	12.3	8.20–16.1	
Hyperlipidemia	10.6	8.39–14.3	0.457
No COPD^b^	11.0	8.45–15.9	
COPD	10.4	8.07–15.3	0.695
COX-2			
No current smoking	10.1	4.68–15.8	
Current smoking	10.3	5.34–17.6	0.604
No renal insufficiency	9.27	5.25–18.7	
Renal insufficiency	10.9	5.30–14.6	0.678
No hypertension	8.57	5.09–16.7	
Hypertension	10.4	5.70–16.0	0.695
No diabetes mellitus	9.27	4.70–15.9	
Diabetes mellitus	11.6	6.90–24.7	0.188
No hyperlipidemia	9.28	5.36–16.5	
Hyperlipidemia	10.6	5.09–15.8	0.990
No COPD^b^	10.1	5.20–16.5	
COPD	10.6	5.60–15.0	0.889
mPGES-1			
No current smoking	2.42	1.16–4.08	
Current smoking	3.46	2.33–4.95	0.009
No renal insufficiency	2.69	1.24–3.82	
Renal insufficiency	2.69	1.90–4.67	0.245
No hypertension	2.87	1.74–3.67	
Hypertension	2.66	1.48–4.46	0.735
No diabetes mellitus	2.78	1.50–4.26	
Diabetes mellitus	2.47	1.20–4.36	0.606
No hyperlipidemia	2.66	2.06–3.65	
Hyperlipidemia	2.76	1.20–4.51	0.798
No COPD^b^	2.39	1.16–4.09	
COPD	2.90	2.35–4.75	0.06

^a^Mann-Whitney Rank Sum Test.

^b^Chronic obstructive pulmonary disease.

**Table 3:** *χ* ^2^ analysis of risk factors.

Variable	COPD	CS	DM	HL	RI
HTN	*χ* ^2^ = 1.494	*χ* ^2^ = 3.371	*χ* ^2^ = 1.801	*χ* ^2^ = 0.0318	*χ* ^2^ = 4.448
	*P* = 0.222	*P* = 0.066	*P* = 0.180	*P* = 0.859	*P* = 0.035

COPD		*χ* ^2^ = 8.464	*χ* ^2^ = 0.0006	*χ* ^2^ = 0.082	*χ* ^2^ = 9.952
		*P* = 0.004	*P* = 0.981	*P* = 0.775	*P* = 0.002

CS			*χ* ^2^ = 0.0326	*χ* ^2^ = 0.0009	*χ* ^2^ = 1.223
			*P* = 0.857	*P* = 0.976	*P* = 0.269

DM				*χ* ^2^ = 2.55	*χ* ^2^ = 0.04
				*P* = 0.110	*P* = 0.842

HL					*χ* ^2^ = 0.003
					*P* = 0.957

HTN: arterial hypertension; COPD: chronic occlusive pulmonary disease; CS: current smoking; DM: diabetes mellitus; HL: hyperlipidemia; RI: renal insufficiency.

**Table 4:** Plasma levels of PGE_2_ in the AAA samples stratified by cardiovascular risk factors. The number of patients in every group is indicated in Table 1. Plasma levels of PGE_2_ are expressed as pg/mL.

	Median	25th–75th percentile	*P* value versus no-factor^a^
Plasma-PGE_2_			
No current smoking	55.9	39.3–71.1	
Current smoking	68.0	57.1–119.5	0.008
No renal insufficiency	58.4	44.0–71.4	
Renal insufficiency	59.4	39.3–104.3	0.786
No hypertension	70.1	53.0–95.2	
Hypertension	56.2	39.4–77.2	0.140
No diabetes mellitus	56.5	38.6–94.9	
Diabetes mellitus	60.7	55.8–68.8	0.294
No hyperlipidemia	55.8	44.5–101.9	
Hyperlipidemia	60.7	41.2–72.0	0.947
No COPD^b^	59.4	43.3–74.3	
COPD	57.1	43.5–115.9	0.807

^a^Mann-Whitney rank sum test.

^b^Chronic occlusive pulmonary disease.

**Table 5:** Transcript levels of PGE receptors in the AAA samples stratified by cardiovascular risk factors. The number of patients in every group is indicated in Table 1. Data are expressed as relative to *β*-actin x1000.

	Median	25th–75th percentile	*P* value versus no-factor^a^
EP-2			
No current smoking	3.98	2.53–8.63	
Current smoking	4.84	2.42–7.46	0.811
No renal insufficiency	3.98	2.66–9.05	
Renal insufficiency	4.49	2.41–7.72	0.653
No hypertension	4.58	2.42–8.22	
Hypertension	4.10	2.56–8.41	0.946
No diabetes mellitus	3.98	2.45–7.24	
Diabetes mellitus	5.27	2.88–23.6	0.115
No hyperlipidemia	3.91	2.38–6.60	
Hyperlipidemia	5.31	2.82–12.0	0.068
No COPD^b^	3.98	2.51–9.82	
COPD	4.95	2.60–7.46	0.846
EP-3			
No current smoking	2.80	1.30–6.18	
Current smoking	3.41	2.16–6.32	0.202
No renal insufficiency	2.68	1.47–4.74	
Renal insufficiency	3.36	1.94–8.02	0.172
No hypertension	3.12	1.98–5.74	
Hypertension	3.06	1.45–6.30	0.484
No diabetes mellitus	3.18	1.87–5.67	
Diabetes mellitus	1.94	0.64–10.4	0.305
No hyperlipidemia	3.47	1.82–7.19	
Hyperlipidemia	2.54	1.46–5.32	0.288
No COPD^b^	3.16	1.49–6.05	
COPD	2.95	1.92–6.21	0.853
EP-4			
No current smoking	4.29	3.24–6.49	
Current smoking	5.93	4.87–8.92	0.007
No renal insufficiency	4.63	3.47–6.25	
Renal insufficiency	5.70	3.45–7.72	0.259
No hypertension	4.89	3.94–7.72	
Hypertension	4.93	3.45–6.61	0.394
No diabetes mellitus	4.92	3.48–6.66	
Diabetes mellitus	4.71	1.82–7.96	0.678
No hyperlipidemia	4.90	3.26–6.61	
Hyperlipidemia	4.91	3.51–7.32	0.678
No COPD^b^	4.90	3.52–6.57	
COPD	5.20	2.41–7.61	0.896

^a^Mann-Whitney rank sum test.

^b^Chronic obstructive pulmonary disease.

## Abbreviations

AAA: Abdominal aortic aneurysm
HTN: Arterial hypertension
COPD: Chronic occlusive pulmonary disease
CS: Current smoking
eGFR: Estimated glomerular filtration rate
HL: Hyperlipidemia
MVEC: Microvascular endothelial cells
RI: Renal insufficiency
VSMC: Vascular smooth muscle cells.
